# Chevron versus scarf osteotomy for 1-2 intermetatarsal reduction in the surgical treatment of hallux valgus: a systematic review and meta-analysis

**DOI:** 10.1186/1757-1146-4-S1-O44

**Published:** 2011-05-20

**Authors:** Simon E Smith, Karl B Landorf, Hylton B Menz

**Affiliations:** 1 Department of Podiatry, Faculty of Health Sciences, La Trobe University, Bundoora, Victoria, Australia; 2 Musculoskeletal Research Centre, Faculty of Health Sciences, La Trobe University, Bundoora, Victoria, Australia

## Background

Surgical correction for hallux valgus (HV) has been demonstrated to be effective compared with watchful waiting and orthotic therapy, and improves the quality of life of individuals with HV. The severity of the HV deformity generally dictates the type of procedure choice, and it is generally considered that the scarf osteotomy affords a greater reduction of the 1-2 intermetatarsal angle (IMA) than the chevron osteotomy. The objective of this study was to compare the radiographic angular correction of the 1-2 IMA for the chevron versus the scarf osteotomy.

## Methods

A systematic review and meta-analysis was conducted. The following databases were searched to identify English language studies evaluating the chevron and scarf osteotomy: Medline, Embase (Ovid), CINAHL (EBSCO Host), the Cochrane Database of Systematic Reviews and Cochrane Central Register of Controlled Clinical Trials. No date restrictions, previous to November 2010, were applied. Additional hand and electronic content searches of orthopaedic and foot-related journals and text books were performed. Randomised controlled trials, and prospective and retrospective cohort studies were included in the analysis. The mean and standard deviation for pre- and post-operative 1-2 IMA for included studies were entered into Review Manager for analysis. The pooled mean difference (in degrees) and 95% confidence intervals (95% CIs) for the 1-2 IMA were compared between categories (i.e. chevron versus scarf osteotomy).

## Results

There were 24 studies meeting the inclusion criteria for the chevron osteotomy and 8 studies for the scarf osteotomy, involving a total of 1303 patients. The pooled mean difference and 95% CIs for the chevron and scarf osteotomies were 5.30±0.21 degrees (5.09, 5.51) and 6.21±0.51 degrees (5.70, 6.72), respectively.

## Conclusions

The scarf osteotomy produces a marginally greater reduction in the 1-2 IMA compared with the chevron osteotomy. However, the included studies were of moderate to poor methodological quality, somewhat limiting the confidence of this result. In addition, our review did not analyse the post-operative clinical outcomes of these two procedures. There is therefore a need for more well designed prospective cohort and randomized controlled trials to compare the angular correction and clinical outcomes of the chevron and scarf osteotomy.

